# Uncertainty in clinical practice – an interview study with Swedish GPs on patients with sore throat

**DOI:** 10.1186/s12875-016-0452-9

**Published:** 2016-05-18

**Authors:** Malin Andre, Hedvig Gröndal, Eva-Lena Strandberg, Annika Brorsson, Katarina Hedin

**Affiliations:** Department of Medicine and Health Sciences, Family Medicine, Linköping University, Linköping, Sweden; Department of Public Health and Caring Sciences, Family Medicine and Preventive Medicine, Box 564, 75122 Uppsala, Sweden; Department of Sociology, Uppsala University, Uppsala, Sweden; Lund University, Department of Clinical Sciences, Family Medicine, Malmö, Sweden; Blekinge Centre of Competence, Blekinge County Council, Karlskrona, Sweden; Center for Primary Health Care Research, Skåne Region, Malmö, Sweden; Department of Research and Development, Kronoberg County Council, Växjö, Sweden

**Keywords:** Uncertainty, General practitioners, Sore throat, Guideline, C-reactive protein, Qualitative research

## Abstract

**Background:**

Uncertainty is inevitable in clinical practice in primary care and tolerance for uncertainty and concern for bad outcomes has been shown to vary between physicians. Uncertainty is a factor for inappropriate antibiotic prescribing. Evidence-based guidelines as well as near-patient tests are suggested tools to decrease uncertainty in the management of patients with respiratory tract infections. The aim of this paper was to describe strategies for coping with uncertainty in patients with pharyngotonsillitis in relation to guidelines.

**Methods:**

An interview study was conducted among a strategic sample of 25 general practitioners (GPs).

**Results:**

All GPs mentioned potential dangerous differential diagnoses and complications. Four strategies for coping with uncertainty were identified, one of which was compliant with guidelines, “Adherence to guidelines”, and three were idiosyncratic: “Clinical picture and C-reactive protein (CRP)”, “Expanded control”, and “Unstructured”. The residual uncertainty differed for the different strategies: in the strategy “Adherence to guidelines” and “Clinical picture and CRP” uncertainty was avoided, based either on adherence to guidelines or on the clinical picture and near-patient CRP; in the strategy “Expanded control” uncertainty was balanced based on expanded control; and in the strategy “Unstructured” uncertainty prevailed in spite of redundant examination and anamnesis.

**Conclusion:**

The majority of the GPs avoided uncertainty and deemed they had no problems. Their strategies either adhered to guidelines or comprised excessive use of tests. Thus use of guidelines as well as use of more near-patient tests seemed associated to reduced uncertainty, although the later strategy at the expense of compliance to guidelines. A few GPs did not manage to cope with uncertainty or had to put in excessive work to control uncertainty.

**Electronic supplementary material:**

The online version of this article (doi:10.1186/s12875-016-0452-9) contains supplementary material, which is available to authorized users.

## Background

Uncertainty is inevitable in clinical practice and perhaps most prominent in work in primary care [1], where patients consult with a wide range of problems, with symptoms in early phases of disease and with problems of life which blur with medicinal problems. Three types of uncertainty have been identified: technical, due to inadequate scientific information; personal, from not knowing the patients’ wish; and conceptual, from problems in applying abstract data to concrete situations [2]. The last decades’ focus on EBM and guidelines sought to remedy one of the identified factors, the insufficient knowledge. Uncertainty is one factor explaining variation in practice and unnecessary use of resources, i.e. laboratory tests in primary care [3]. One of the few studies characterizing individual GPs’ diagnostic strategy in relation to stress from uncertainty showed a positive correlation between anxiety due to uncertainty and diagnostic activity [4, 5].

The physicians’ emotional reactions to uncertainty are described as stress and concern about bad outcomes [1]. Tolerance for uncertainty has been shown to vary between physicians. Experienced physicians tolerate uncertainty better than younger ones and males better than females [1, 6]. A strategy for tolerance is avoidance of or reluctance to disclose uncertainty, learnt during the education of the young doctors [7].

Uncertainty is also discussed as a factor for unnecessary use of antibiotics, which is a threat to modern healthcare. In one recent systematic review, diagnostic uncertainty was found to be the principal indirect fact for inappropriate antibiotic prescribing [8], while fear of possible complications was identified in another [9]. GPs who felt greater discomfort in the face of uncertainty seemed to prescribe antibiotics more often [10]. Recent years’ achievements in research concerning infections have resulted in expanded evidence relevant for the primary care population and evidence-based guidelines are published in many Western countries for common infections in primary care. As another suggested tool to decrease uncertainty and increase appropriate antibiotic prescribing, near-patient tests have been introduced in primary care in several Western countries [8]. Thus important means to reduce uncertainty are now used for common infections in primary care.

In Sweden guidelines for patients with sore throat (pharyngotonsillitis) from 2001 were updated in 2012 [11, 12]. The guidelines aim at a rational use of antibiotics by identifying patients likely to benefit from antibiotic treatment. It is based on reliable evidence that sore throat is a self-limiting disease and the benefit of antibiotics primarily is to relieve symptoms in patients with an infection with Streptococcus group A (GAS). The guidelines recommend the use of the clinical Centor criteria (absence of cough, fever >38.5 °C, tender lymphadenitis and tonsillar coating) to increase the prevalence of patients with GAS infection and thereafter to verify infection with GAS using the near-patient test Rapid Antigen Detection Test for GAS (RADT). There is ample evidence that it is impossible to distinguish between virus and GAS with the naked eye [13]. At the time of the study the guidelines recommended using RADT to verify the presence of GAS when ≥2 Centor criteria were present and antibiotic treatment when the test was positive. The use of C-reactive protein (CRP) test is not recommended in either version of the guidelines as there is limited evidence of benefit in patients with sore throat [11, 12]. Near-patient RADT and CRP in primary care have been used in the Nordic countries for about 25 years. Relevant differential diagnoses (DD) and complications are listed in the guidelines [11, 12].

Studies of the actual behaviour of physicians as well as descriptions of what coping with uncertainty entails have been called for [1]. More complex problems create more uncertainty for the GP than straightforward problems. Managing patients with a sore throat has been used as an example of an uncomplicated clinical problem [1]. However, even this clinical situation contains elements of uncertainty and concern for bad outcomes. In a recent interview study with GPs concerning the management of patients with a sore throat, bad outcomes (potentially threatening DD and complications) were identified as a constant theme for all respondents [14, 15]. Therefore the aim of this paper was to describe strategies for coping with uncertainty in patients with pharyngotonsillitis in relation to guidelines,

## Methods

### Study design

A strategic sample of GPs was chosen with regard to sex, age, educational background, and working experience, urban or rural Primary Health Care Centres, and from areas with high and low antibiotic prescribing from five different counties in Sweden. In total 25 GPs were interviewed individually about their management of sore throat in early 2012 [14]. GPs were recruited in different ways; some were known by the authors, others were recruited by the manager of the health centre. No incentive was used. We used a semi-structured interview guide with open-ended questions. Topics for the interview were: 1) description of the management of patients with sore throat, 2) difficulties in management, 3) near-patient tests used and 4) knowledge of and attitude to guidelines 5) concerns (“What are you worried about?”). Four of the authors conducted half-hour long face to face interviews in the summer and early autumn of 2012 in a place chosen by the interviewed GP. The interviews were voice-recorded and transcribed verbatim. To ensure consistency, the interviewers read each other’s interviews continuously.

### Data analysis

The analysis started with three of the authors (HG, MA and KH) using a template-based analysis [16]. The template included attitude to the guideline, and adherence to the guideline for the diagnosis of GAS. The GP who knew the Centor criteria and said they used RADT according to guidelines was classified as adherent towards the current guidelines for the diagnosis of GAS. The interviews were analysed with regard to the possible DD and complications and whether the GPs described themselves as fearful or not (i.e. expressions that indicated fear uttered in direct connection with DD and complications). The DD and complications described were compared to those mentioned in the Swedish guidelines from 2001 to 2012. Then the interviews were analysed with regard to whether the GPs said it was possible to identify potential DDs and complications.

In the next step all interviews were classified in accordance with this template-based analysis and sorted into groups. The sorted interviews were once more analysed and the coping strategies identified and coded [16]. After that the expressions of the residual uncertainty were identified for each coping strategy. The analysis was performed manually without any software tools.

The other authors read all the material, reflected, commented and confirmed that they contained data supporting the findings of this study. For more details see the Additional file 1.

### Ethical considerations

According to Swedish legislation, ethical approval from the regional ethical review board was not needed for this study since it was part of a quality improvement activity and no patients were involved. The study was, however, approved by the local ethics committee in Kronoberg County 8/2012. All participants gave their informed consent and were informed that participation was voluntary and that they could withdraw at any time, that all data were handled confidentially and that the results would be presented in a non-identifiable way.

## Results

The 25 participating GPs were between 33–64 years of age, half were women and 22 GPs had a working experience ≥5 years [14].

Four strategies for coping with uncertainty were identified, one of which was compliant with guidelines, “Adherence to guidelines”, and three were idiosyncratic: “Clinical picture and CRP”, “Expanded control”, and “Unstructured”. The residual uncertainty differed for the different strategies: in the strategy “Adherence to guidelines” and “Clinical picture and CRP” uncertainty was avoided, based either on adherence to guidelines or on clinical picture and near-patient CRP; in the strategy “Expanded control” uncertainty was balanced based on expanded control; and in the strategy “Unstructured” uncertainty prevailed in spite of redundant examination and anamnesis (Table 1).

**Table 1:** Physician management of patients with sore throat in relation to strategy for coping with uncertainty

	Uncertainty and coping strategies			
	Uncertainty avoided through adherence to guidelines	Uncertainty avoided through clinical picture and CRP	Uncertainty balanced through expanded control	Unstructured and uncertainty prevails
Attitude to guidelines	Guidelines trusted	Guidelines of little concern for clinical practice	Guidelines of little concern for clinical practice	Guidelines trusted
Diagnosis of GAS	Adherent to guidelines	Non-adherent to guidelines	Non-adherent to guidelines	Non-adherent to guidelines
Differential diagnoses (DD) and complications	DD and complications retrieved in guidelines	DD and complications also retrieved outside guidelines	DD and complications retrieved in guidelines	DD and complications also retrieved outside guidelines
Fear of bad outcomes	No fear described	No fear described	Fear described	Fear described
DD and complications possible to identify	DD possible to identify	DD possible to identify	DD possible to identify	DD not possible to identify

### Attitudes towards guidelines

The attitude to the guidelines differed. GPs using the strategy “Adhering to guidelines” and “Unstructured” trusted them (Table 2 Quotation A). Although the GPs in the other strategies knew of the guidelines, the guidelines seemed to be of little concern for the GPs’ clinical practice (Quotation B).

**Table 2:** Meaning units, categories and themes

Meaning units	Category	Themes
(Quotation A)	Guidelines trusted	Attitudes to guidelines
“and I think that’s nice, because I…you know, I think that…they think I look young or small and a girl and all that, and what would you know about it, then it’s very nice to be able to say that it’s not just me who thinks something, it agrees with something that is generally recommended” (Interview 12, p. 17)		
(Quotation B)	Guidelines of little concern for practice	
“The guidelines are always right, I think, but we must see the patient and then use those guidelines on the patient, so it must…we must use our brain. I feel it’s not as simple as to…just to take the guidelines and then…no, you must make the right diagnosis and then you can use guidelines” (Interview 14, p. 20)		
(Quotation C)	Adherent to guidelines	Diagnosis of GAS
“If they have had a fever at least 3 days and absence of a cold and cough then I would always prescribe a RADT.” (Interview 17)		
Quotation D	Non-adherent to guidelines	
“If they then have what’s typical for me, that they have a swollen throat with a really, you know, nasty throat, and lymph glands on the throat and just throat symptoms and fever, then I tend to think like this, yes, this is classic tonsillitis, then I don’t take any tests.” (Interview 2, p. 2)		
Quotation E		
“But sometimes I’m uncertain, and then I take and I see that tonsillitis is… the patient has enlarged tonsils and redness, but if there’s no furring or anything, then I can take Strep-A.” (Interview 22, p. 5)		
(Quotation F)	No fear	Fear of bad outcomes
“No I [am not afraid]. It could be both a streptococcus infection and mononucleosis, but then they won’t get well, they will return and then you’ll check for mononucleosis.” (Interview 19)		
(Quotation G)	Fear	
“I have seen some examples of that, which are frightening, for instance these lateral…whatever it’s called. So I want to rule that out. And then I think that…you know, this thing with incipient peritonsillitis you have to be a bit observant about.” (Interview 7, p. 10)		
(Quotation H)	Not possible to identify	DD and complications possible to identify
“That’s what I find most difficult because you have to look down there properly, I think, in the larynx and look at the vocal cords and dare…so to speak, someone who’s hoarse, to see that it’s not something malignant, that you miss something dangerous, I find that really difficult. You often end up in the situation that you’ve seen a little, but not enough to rule out completely that it could be dangerous.” (Interview 6, p. 2)		
(Quotation I)	Possible to identify	
“When they say, for example, that the find it hard to open wide, I think that often they come here and sit in with the nurse and the nurse is a bit uncertain, then I usually go in and take a look and then you can see. And then it’s mostly a matter of a…is it a sore throat or is it actually peritonsillitis.” (Interview 12, p. 15)		
(Quotation J)	Adherence to guidelines	Coping strategy
“I think it’s good that we have these Centor criteria, you know, rules, because it’s…well, if you follow them it’s not difficult.” (Interview 17, p. 7)		
(Quotation K)	Clinical picture and CRP	
“In my view the clinical assessment is the most important” (Interview 14, p. 6)		
(Quotation L)		
“Then I take CRP too, to know whether it’s over 50 or 60, then you think it’s something more bacterial than virus.” (Interview 22, pp. 7–8)		
(Quotation M)	Expanded control	
“But I am still very careful to look down the throat, I must say that, and perhaps you feel a greater need to check with the years, when you’ve seen that there can be things there.” (Interview 7, pp. 3,9)		
Quotation N)	Unstructured	
“Well, I ask the patient of course, how long, how much it hurts, where it hurts, if they have difficulty swallowing, if they have a temperature, if it’s the first time, if they recognize it or if it’s something they’ve have before, if they’ve had it recently and had the same symptoms and if they’ve been to the doctor and had treatment for it, if there’s anyone in the family who’s been sick, or that they’ve met…that that person has met someone who has had the same illness. That means I’ve sort of covered a bit there. And then I ask what they work at, I ask if they have children, I ask general questions and then the usual history taking… I ask if they have earache or some other respiratory tract symptoms that might be connected to it, you know, earache or a cough or a cold, that kind of thing, sore joints…you know, the most usual things. Well, then I examine the patient so that I look in the ears, nose, throat, right, and feel the lymph glands on the throat and often, in fact, I listen to the lungs too, since they’ve come for a consultation anyway.” (Interview 2, p. 1)		

### Diagnosis of GAS

GPs using the strategy “Adherence to guidelines” could recall the Centor criteria, stated use of RADT when at least 2 Centor criteria were present and prescribed antibiotics only when RADT was positive (Quotation C). GPs using the other strategies stated that a patient presenting a typical picture for GAS was prescribed antibiotics without RADT, which instead was used when in doubt (Quotations D and E).

### Differential diagnoses and complications

All interviewed GPs mentioned potential dangerous DDs and possible complications (Table 3). In strategy “Adherence to guidelines” and “Expanded control” DD and complications mentioned were retrieved in the guidelines. In the strategy “Clinical picture and CRP” and “Unstructured” the GPs added many different differential diagnoses, also outside the Swedish guidelines. Moreover, the GPs underlined the possibility of an unspecified bacterial infection, spread in the body, which might present itself by the patient feeling sick, a bacterial smell or raised CRP.

**Table 3:** Differential diagnoses and complications mentioned by the interviewed GPs

	Mentioned differential diagnoses and complications of GAS
Retrieved in guidelines	Peritonsillitis
	Mononucleosis
	Epiglottitis
	Tongue base tonsillitis
	Streptococcus group C and G
	Anaerobic infection
	Viral infection
	Glomerulonephritis
	Cancer
	Something further down in the throat
Not retrieved in guidelines	Pneumonia
	Bacterial infection
	Sinusitis
	Infection with *Hemophilus Influenzae*
	Temporalis arteritis
	Diphtheria
	Tularemia
	Spread infection
	Problems with the stomach
	Foreign body
	Secondary infection
	Infection with staphylococcus

### Fear of differential diagnoses and complications

The vast majority of GPs using strategies “Adherence to guidelines” and “Clinical picture and CRP” expressed no fear when talking about DDs and complications (Table 2, Quotation F). However, in strategy “Expanded control” and “Unstructured”, the conditions were described as daunting (Quotation G).

### DD and identifiable complications

GPs using the strategy “Unstructured” stated that it was impossible to identify possible DDs and complications (Quotation H), while GPs using the other strategies found it conceivable (Quotations F and I).

### Coping strategies and resulting uncertainty

Four different strategies for coping with uncertainty were identified, reflecting variations in management in relation to attitudes to guidelines, diagnosis of GAS, fear, and possible differential diagnoses and complications (Table 1). The resulting clinical uncertainty differed as well. A third of the GPs used the strategy of trusting and adhering to guidelines. In this strategy, “Adherence to guidelines”, uncertainty was avoided. The DD and complications (mentioned without fear) were described as possible to diagnose with Centor criteria, clinical examination and RADT, if necessary with wider laboratory analysis chiefly for mononucleosis (Table 2, Quotations F, I and J). In the strategy “Clinical picture and CRP”, used by slightly more than half of the GPs, the crucial point stressed was the clinical assessment of the GP (Quotations K). CRP was used to differentiate between viral and bacterial infection on the presumption that an unspecified bacterial infection indicated the need for antibiotics (Quotation L). DD and complications, also outside those retrieved in the guidelines, were mentioned without fear and uncertainty was removed. Safety netting (planning for new contact if symptoms did not resolve) was mentioned by GPs in both the strategies “Adherence to guidelines” and “Clinical picture and CRP”.

In strategy “Expanded control” a few GPs described how experience with earlier patients had led them to extend the examination (for example both clinical and laboratory, i.e. culture, mirror examination of the larynx) more than recommended (Quotation M). Although the DD and complications were described as frightening (Quotation G), it was possible to control for them by this expanded examination. Finally, in the strategy “Unstructured” a couple of GPs told of a redundant anamnesis and examination with no clear focus (Quotation N). In spite of this the GPs expressed the impossibility to control for the DD and complications (Quotation H), which were expressed as frightening.

## Discussion

In this interview study with 25 strategically selected GPs from different parts of Sweden we explored the management of patients with sore throat and identified the residual uncertainty. Four strategies for coping with uncertainty were identified, one of which was “Adherence to guidelines”, and three were idiosyncratic: “Expanded control”, “Clinical picture and CRP” and “Unstructured”. The residual uncertainty differed for the different strategies: in the strategy “Adherence to guidelines” and “Clinical picture and CRP” uncertainty was avoided, in “Expanded control” fear of bad outcomes was balanced, but in the strategy “Unstructured” uncertainty prevailed and the GP was out of control. To our knowledge no earlier study has explored strategies for coping with uncertainty in relation to a clinical problem in primary care. This study gives new insights into how the inherent uncertainty in clinical practice is managed in relation to guidelines in patients with sore throat in primary care.

A maximum variety sampling of participants from different parts of Sweden was achieved. Also, none of the invited GPs declined to be interviewed. The analysis with both inductive and deductive elements required iterative close readings of the interviews, which increases the validity of the results [16].

One weakness of the study is that four different interviewers may have decreased the reliability of the interviews. Still, all used the interview guide and the interviews were read and discussed continuously in order to reach consistency. Four of the interviewers had been involved in implementing the sore throat guidelines and thus could have been perceived as experts on the informants, which may have biased the interviews.

In this study we used expressions of fear in connection with DD or complications as a measure of concern for bad outcomes. In the interview manual questions of difficulties and concerns in patients with sore throat were asked but did not include direct questions about how GPs tolerated uncertainty. Earlier studies on physicians’ reactions to uncertainty used the “Physician reaction to uncertainty” scale questions, which besides concerns for bad outcomes also measures anxiety due to uncertainty and reluctance to disclose uncertainty to patients and physicians [1]. Thus the result of our study is not directly comparable with studies where the scale was used. However, this qualitative study deepens the understanding of the clinical practice with regard to managing uncertainty in primary care.

Near-patient tests (RADT and CRP) have been used for more than two decades in the Nordic countries, and the generalizability of this study might seem low to countries where the test is not in use. However, the test is being introduced in more countries, and therefore the result of our study might be relevant also for other countries. Finally, no observation of actual behaviour was included in the study. A link between what you say and what you do may not always be straightforward.

Scientific medicine is described as a symbolic system for coping with fears and uncertainties in medicine [1]. For the absolute majority of GPs uncertainty was avoided and our study thus confirms earlier findings [1, 5, 7]. However, the result may have differed if a more complex patient problem been studied. If the diagnosis seems unclear, more complications remain possible, thus diagnostic uncertainty and fear of possible complications seem to be related [8]. One consistent theme in studies of uncertainty is fear of personal inadequacy and failure. Young physicians must learn to control uncertainty or to be paralysed by it [1]. Denial and avoidance of uncertainty is a strategy to tolerate uncertainty together with physicians’ propensity to resolve uncertainty by action rather than inaction [1, 5].

For patients as common as those with a sore throat, GPs have to develop a strategy that decreases uncertainty to be able to last in their work. A greater stress of uncertainty was indicated by the GPs using both the strategy “Expanded control”, where uncertainty was managed by an excessive workload, and the strategy “Unstructured”, where uncertainty was not controlled. Personality traits seem related to diagnostic reasoning, and in one study of GPs the personality trait Neuroticism was associated with more anxiety due to uncertainty [17]. Stress caused by uncertainty correlates with work satisfaction and a higher risk of burnout [18]. Thus the GPs using the strategies “Expanded control” and “Unstructured” seem at risk of not coping with work in the long run.

All GPs knew of the guidelines but only one third of the interviewed GPs used the strategy “Adherence to guidelines” and felt no uncertainty. In the light of the systematic work carried out in Sweden with distributed guidelines in printed short version and regular outreach visits to all health centres this looks like a small number [19]. However, as the guideline did not include a dominating conception among GPs using the strategy “Clinical picture and CRP”, namely unspecified bacterial infection, it was not perceived relevant for their clinical practice. A similar mismatch between the content of the guideline and the conception of GPs might explain the results from the German study of GPs by Schneider et al. The use of guidelines did not contribute to understand the GPs strategies to manage uncertainty, which the authors suggest might be due to the gap between guidelines and the actual clinical practice [4].

In this study an excess of laboratory tests compared to the guidelines was described in all strategies except “Adhering to guidelines”. In the strategy “Clinical picture and CRP” the result of the CRP tests induced certainty in the GPs. However, it could be said that this was a false sense of certainty. There are only two small studies describing CRP in patients with sore throat, and therefore the evidence to interpret the result lacks validity [20, 21]. Perhaps this delight in CRP is an expression of “Our stubborn quest for security” as Kassier put it [22]. He calls for us to weed out tests that are ineffective or poorly predictive.

Tolerance of uncertainty seems to affect test-ordering behaviour [3]. Increase of diagnostic activity was found in the strategy “Expanded control” as in an earlier study of German GPs [4]. Risk-adverse GPs in France more often used RADT and more often prescribed antibiotics when RADT was not used compared to risk-tolerant GPs [23]. In our study no measures of tolerance of uncertainty were included.

GPs have to cope with uncertainty, especially in common patient problems like sore throat. A few GPs did not manage to do so or had to put in excessive work to control uncertainty with risk of lower work satisfaction and burnout. For these GPs reflective writing or case-based reasoning might be a remedy [12, 24, 25]. The majority of the interviewed GPs, however, had no feeling of uncertainty when dealing with patients with a sore throat. Although the greater part of these GPs used a strategy, which did not adhere to guidelines, they deemed that they did not have any problem. To change habits and preconceptions in situations like that is not easy. Addressing current conceptions and fears of bad concerns in new or revised guidelines might be one way forward. Small group discussions together with feedback and benchmarking of performance indicators might be another way to go.

## Conclusions

The majority of the GPs avoided uncertainty and deemed they had no problems. Their strategies either adhered to guidelines or comprised excessive use of tests. Thus use of guidelines as well as use of more near-patient tests seemed associated to reduced uncertainty, although the later strategy at the expense of compliance to guidelines. A few GPs did not manage to cope with uncertainty or had to put in excessive work to control uncertainty.

### Availability of data and materials

All the data supporting the findings is contained within the manuscript.

## Additional file

Additional file 1: COREQ checklist. (DOCX 24 kb)

## References

[1] Gerrity M, Earp JA, DeVellis RF, Light D (1992). Uncertainty an professional work: perceptions of physicians in clinical practice. Am J Sociol.

[2] Beresford EB (1991). Uncertainty and the shaping of medical decisions. Hastings Cent Rep.

[3] van der Weijden T, van Bokhoven MA, Dinant GJ, van Hasselt CM, Grol RP (2002). Understanding laboratory testing in diagnostic uncertainty: a qualitative study in general practice. Br J Gen Pract.

[4] Schneider A, Lowe B, Barie S, Joos S, Engeser P, Szecsenyi J (2010). How do primary care doctors deal with uncertainty in making diagnostic decisions? The development of the ‘Dealing with Uncertainty Questionnaire’ (DUQ). J Eval Clin Pract.

[5] Katz J (1984). Why doctors don't disclose uncertainty. Hastings Cent Rep.

[6] Nevalainen M, Kuikka L, Pitkala K (2014). Medical errors and uncertainty in primary healthcare: a comparative study of coping strategies among young and experienced GPs. Scand J Prim Health Care.

[7] Atkinson P (1984). Training for certainty. Soc Sci Med.

[8] Teixeira Rodrigues A, Roque F, Falcao A, Figueiras A, Herdeiro MT (2013). Understanding physician antibiotic prescribing behaviour: a systematic review of qualitative studies. Int J Antimicrob Agents.

[9] Lopez-Vazquez P, Vazquez-Lago JM, Figueiras A (2012). Misprescription of antibiotics in primary care: a critical systematic review of its determinants. J Eval Clin Pract.

[10] Brookes-Howell L, Hood K, Cooper L, Little P, Verheij T, Coenen S, Godycki-Cwirko M, Melbye H, Borras-Santos A, Worby P, et al. Understanding variation in primary medical care: a nine-country qualitative study of clinicians’ accounts of the non-clinical factors that shape antibiotic prescribing decisions for lower respiratory tract infection. BMJ Open. 2012;0:e000796. doi:10.1136/bmjopen-2011-000796. 10.1136/bmjopen-2011-000796 PMC4401816 22918670

[11] [Anonymous]: Treatment of pharyngotonsillitis. Medical product agency [In Swedish]. 2001;7/8:44–75.

[12] [Anonymous]. Management of pharyngotonsillitis in ambulatory care - new recommendation. Medical product agency [In Swedish]. 2012;6:18–25.

[13] Aalbers J, O'Brien KK, Chan WS, Falk GA, Teljeur C, Dimitrov BD, Fahey T (2011). Predicting streptococcal pharyngitis in adults in primary care: a systematic review of the diagnostic accuracy of symptoms and signs and validation of the Centor score. BMC Med.

[14] Hedin K, Strandberg EL, Grondal H, Brorsson A, Thulesius H, Andre M (2014). Management of patients with sore throats in relation to guidelines: an interview study in Sweden. Scand J Prim Health Care.

[15] Grondal H, Hedin K, Strandberg EL, Andre M, Brorsson A (2015). Near-patient tests and the clinical gaze in decision-making of Swedish GPs not following current guidelines for sore throat - a qualitative interview study. BMC Fam Pract.

[16] Crabtree M, Miller W (1992). Doing qualitative research.

[17] Schneider A, Wubken M, Linde K, Buhner M (2014). Communicating and dealing with uncertainty in general practice: the association with neuroticism. PLoS One.

[18] Cooke GP, Doust JA, Steele MC (2013). A survey of resilience, burnout, and tolerance of uncertainty in Australian general practice registrars. BMC Med Educ.

[19] Molstad S, Erntell M, Hanberger H, Melander E, Norman C, Skoog G, Lundborg CS, Soderstrom A, Torell E, Cars O. Sustained reduction of antibiotic use and low bacterial resistance: 10-year follow-up of the Swedish Strama programme. Lancet Infect Dis. 2008;8(2):125–32. 10.1016/S1473-3099(08)70017-3 18222163

[20] Hjortdahl P, Melbye H (1994). Does near-to-patient testing contribute to the diagnosis of streptococcal pharyngitis in adults? [see comments]. Scand J Prim Health Care.

[21] Gulich MS, Matschiner A, Gluck R, Zeitler HP (1999). Improving diagnostic accuracy of bacterial pharyngitis by near patient measurement of C-reactive protein (CRP)[comment]. Br J Gen Pract.

[22] Kassirer JP (1989). Our stubborn quest for diagnostic certainty. A cause of excessive testing. [see comments]. N Eng J Med.

[23] Michel-Lepage A, Ventelou B, Nebout A, Verger P, Pulcini C (2013). Cross-sectional survey: risk-averse French GPs use more rapid-antigen diagnostic tests in tonsillitis in children. BMJ Open.

[24] Nevalainen MK, Mantyranta T, Pitkala KH (2010). Facing uncertainty as a medical student--a qualitative study of their reflective learning diaries and writings on specific themes during the first clinical year. Pat Educ Couns.

[25] Sommers LS, Launer J (2013). Clinical uncertinties in primary care. The challenge of collaborative engagement.

