# Supplementary material for: Uncertainty in clinical practice – an interview study with Swedish GPs on patients with sore throat
Source: BMC Fam Pract. 2016 May 18;17:56. doi: 10.1186/s12875-016-0452-9 (PMC4870808; doi:10.1186/s12875-016-0452-9)
Supplement: Additional file 1: — COREQ checklist. (DOCX 24 kb) [file 12875_2016_452_MOESM1_ESM.docx]

| Personal Characteristics |  |  |
| --- | --- | --- |
| 1. | Interviewer/facilitator | The interviews were conducted by Malin André (MA), Annika Brorsson (AB), Katarina Hedin (KH) and EvaLena Strandberg (ELS). |
| 2. | Credentials | The credentials for the researchers are: MA: GP and PhD, AB: GP and PhD, KH: GP and PhD, ELS: MSc and PhD, HG: PhD candidate |
| 3. | Occupation | MA: research project manager in primary care, AB: GP, HG: PhD candidate, KH: Research and development manager, ELS: Research and Development leader and project manager |
| 4. | Gender | All the researchers are females |
| 5. | Experience and training | MA, AB, KH have 15-30 years of experience of work as a GP and research in primary care, ELS has 25 years of experience of research and development in primary care and social work, HG has experience of research as PhD candidate |
| Relationship with participants |  |  |
| 6. | Relationship established | No relationship was established prior to study commencement |
| 7. | Participant knowledge of the interviewer | The participants were informed of the broad aim of the study (management of patients with a sore throat) but not of the specific aim of the present study. All interviewers were known to be involved in primary care research |
| 8. | Interviewer characteristics | The researchers were reported as GP and/or R&D leaders and some were also known to be involved in guideline development |
| **Domain 2: study design** |  |  |
| Theoretical framework |  |  |
| 9. | Methodological orientation and Theory | Qualitative Content analysis underpin the study method |
| Participant selection |  |  |
| 10. | Sampling | The participants were purposive selected |
| 11. | Method of approach | The participants were approached in different ways; face-to-face, telephone, mail, email |
| 12. | Sample size | The participants were 25 in the study |
| 13. | Non-participation | There was no one who declined to participate or dropped out |
| Setting |  |  |
| 14. | Setting of data collection | The data were collected at the workplace of the participants |
| 15. | Presence of non-participants | There was no one else present besides the participants and researchers |
| 16. | Description of sample | All participants were GPs, but differed concerning age, gender, years of working experience, working in urban or rural areas. |
| Data collection |  |  |
| 17. | Interview guide | There were questions provided by the authors, which were pilot tested. |
| 18. | Repeat interviews | No repeat interviews carried out |
| 19. | Audio/visual recording | Audio recording was used to collect the data. |
| 20. | Field notes | No field notes were made during and/or after the interview |
| 21. | Duration | The duration of the interviews were 20-30 minutes |
| 22. | Data saturation | Data saturation was discussed |
| 23. | Transcripts returned | No transcripts were returned to participants for comment and/or correction |
| **Domain 3: analysis and findings**z |  |  |
| Data analysis |  |  |
| 24. | Number of data coders | In the present study three data coders coded the data. All authors discussed the coding until consensus was achieved |
| 25. | Description of the coding tree | The authors provide a description of the coding tree |
| 26. | Derivation of themes | Themes were identified partly in advance and partly derived from the data |
| 27. | Software | No software was used to manage the data |
| 28. | Participant checking | The participants did not provide feedback on the findings |
| Reporting |  |  |
| 29. | Quotations presented | The participant quotations were presented to illustrate the themes / findings. The quotations were identified |
| 30. | Data and findings consistent | There was consistency between the data presented and the findings |
| 31. | Clarity of major themes | The major themes were clearly presented in the findings |
| 32. | Clarity of minor themes | There is no description of diverse cases or discussion of minor themes |
